# Effect of Inoculation with *Lactiplantibacillus plantarum* and Cellulase on the Quality of Mulberry Silage

**DOI:** 10.3390/microorganisms13071437

**Published:** 2025-06-20

**Authors:** Yingchao Sun, Yongcheng Chen, Zhiwei Huo, Guohong Liu, Xiaokai Zheng, Yayin Qi, Chunhui Ma, Fanfan Zhang

**Affiliations:** 1 College of Animal Science & Technology, Shihezi University, Shihezi 832000, China; 17633866730@163.com (Y.S.); 20202013022@stu.shzu.edu.cn (Y.C.); zhengxiaokaiyue@163.com (X.Z.); qiyayin@163.com (Y.Q.); 2 Kashgar Prefecture Animal Husbandry and Veterinary Bureau of Xinjiang Uygur Autonomous Region, Kashi 844000, China; h69896882@163.com; 3 Turpan Institute of Agricultural Sciences, Xinjiang Academy of Agricultural Sciences, Turufan 838000, China; lghdjs@163.com

**Keywords:** silage additives, nutritional quality, bacterial community, microbial inoculation

## Abstract

Lactic acid bacteria (LAB) and cellulase have been used as additives to improve the fermentation quality of mulberry silage. This study investigated the dynamics of fermentation characteristics and bacterial communities during 60-day ensiling through three established treatment groups: Control (no inoculation), *Lactiplantibacillus plantarum* (LP) inoculation as well as combination of *L. plantarum* and cellulase inoculation group (LPC). The results showed that compared with the Control group, the LP and LPC treatments significantly reduced the loss of dry matter, soluble carbohydrates, and crude protein (*p* < 0.05), effectively promoted the accumulation of lactic acid and acetic acid (*p* < 0.05), but significantly elevated ammonia nitrogen (NH_3_-N) production. Inoculation was beneficial to the stability of the bacterial community in mulberry branch and leaf silage because it can maintain a high level of beneficial bacteria (*Lactiplantibacillus*) and inhibit the growth of harmful bacteria (*Escherichia-Shigella*). The combination of the inoculation of *L. plantarum* and cellulase may improve the quality of mulberry branch silage.

## 1. Introduction

Escalating demand for livestock forage necessitates alternative feed resources, such as forest and fruit byproducts. Mulberry (*Morus alba* L.) is a perennial deciduous tree belonging to the Moraceae family. It has high nutritional value and strong environmental adaptability and is widely distributed worldwide [1]. Moreover, mulberry branches and leaves contain substantial crude protein (14.66% DM), minerals, and vitamins [2]. However, high moisture content (50.35% DM) and fiber levels (NDF: 54.30% DM; lignin > 18%) limit its preservation and digestibility [3]. Research has shown that the application of silage fermentation technology to mulberry branches and leaves can transform and decompose macromolecular nutrients in the raw materials, reduce the loss of nutrients, and degrade some crude fiber content [4]. Silage involves anaerobic fermentation by microorganisms, leading to the production of lactic acid, thus inhibiting the growth of pathogenic microorganisms and maintaining the nutritional value of the feed [5]. In addition, the fiber content of mulberry branches and leaves is as high as 60%, and the lignin content of the branches is >18%, leading to poor palatability and digestibility. To improve the quality of mulberry silage, it is necessary to add other feedstuffs that are easily fermented or substances rich in soluble carbohydrates as well as appropriate additives [6]. The co-fermentation and co-storage of enzymes and microorganisms during silage production significantly enhance fermentation [7]. Studies have shown that adding plant lactic acid bacteria and cellulase during silage production can promote the growth of beneficial microorganisms, enhance lactic acid fermentation, and improve the digestibility and nutritional value of silage [8,9].

*Lactiplantibacillus plantarum* (*L. plantarum*) effectively lowers the pH of silage and creates an acidic environment that accelerates fermentation [10]. In this process, the type, quantity, activity, and diversity of lactic acid bacteria will affect the fermentation quality of silage [11,12]. These effects reduce nutrient loss, improve silage fermentation efficiency, and enhance palatability and aerobic stability [13]. Cellulase supplementation degrades fiber to release fermentable sugars, accelerating pH reduction and improving silage preservation [14]. Inoculation with *L. plantarum* further enhances this process by rapidly producing lactic acid, suppressing spoilage microorganisms [10,15]. Combined *L. plantarum*–cellulase treatments improve fermentation in the silage of Rhodesgrass (*Chloris gayana* Kunth.) and Italian Ryegrass (*Lolium multiflorum* Lam.) [16], but the effect of the co-inoculation of *L. plantarum* and cellulase on mulberry silage—notably on bacterial community dynamics—remains unclear.

Therefore, we aim to evaluate the synergistic effects of *L. plantarum* and cellulase inoculation on the fermentation quality and nutritional preservation of mulberry branch silage. In addition, during the silage process, changes in the bacteria community structure and abundance of some microbial may greatly impact fermentation quality [17]. Therefore, it is necessary to characterize the dynamics of microbial communities and their correlations with fermentation parameters during 60-day ensiling. The findings presented here will provide methods and ideas on the source and utilization of roughage resources in animal husbandry.

## 2. Materials and Methods

### 2.1. Silage Prepartion

The leaves and branches of mulberry trees were collected from the Agricultural Science Research Institute in Turpan, Xinjiang (89°20′46″ N, 42°94′79″ E), a region characterized by an arid climate with sandy loam soil, in May 2023. During the peak vegetative growth stage of mulberry trees (approximately 1.5–2 m in height), mechanized harvesting was implemented to collect both branches and foliage, resulting in a biomass ratio of branches to leaves at approximately 1:2. Raw materials were crushed into 1 to 2 cm pieces. The following treatments were applied to the silage: (1) A quantitative urea solution was sprayed onto the surface of the raw material based on its fresh weight prior to ensiling; (2) inoculation with *L. plantarum* strain CICC (China Center of Industrial Culture Collection, Beijing) 23120 and cellulase (Cellulase AP3 from Trichoderma reesei, Solarbio Science & Technology Co., Ltd., Beijing, China; Product No. C8140) was performed (LPC group), with *L. plantarum* performed at a concentration of 5 × 10^6^ colony-forming units of per gram (CFU·g^−1^) fresh weight, cellulase was inoculated at 0.3% fresh weight (enzyme activity ≥ 5000 U·g^−1^ defined as the amount of enzyme liberating 1 μmol glucose per minute from carboxymethyl cellulose at pH 4.8 and 50 °C); (3) inoculation with *L. plantarum* was performed at a concentration of 5 × 10^6^ CFU·g^−1^ of fresh weight (LP group; based on our previous research that the addition of *L. plantarum* alone at 5 × 10^6^ CFU·g^−1^ fresh weight enhanced the quality of paper mulberry silage) [18]; (4) equal volume of sterilized water was added (Control group). Each latic acid bacteria (LAB) strain was enriched in de Man Rogosa Sharpe (MRS) liquid culture medium. After quantifying the LAB strains by plate counting, the bacteria were evenly sprayed over each bag of silage and thoroughly mixed. Vacuum-sealed polyethylene bags measuring 35 × 40 cm were prepared for ensiling, with 20 bags allocated to each group (approximately 1.5 kg each). The bags were stored at ambient temperatures ranging from 24 °C to 40 °C throughout the experiment. The experiment followed a completely randomized design with three treatments and four sampling time points (7, 15, 30, and 60 days). On each sampling day, five bags from each group were opened and samples were collected for parameter measurement.

### 2.2. Mulberry Silage Chemical Components, Fermentation Characteristics, and Microbiological Analyses

The dry matter (DM) content of raw mulberry branches/leaves and silage was determined with modifications [11]: samples were blanched at 105 °C for 30 min in a forced-air oven (DHG-9620A; Shanghai Heheng Equipment Co., Ltd., Shanghai, China), followed by drying at 65 °C to constant weight. Dried samples were ground (DFY-1000D high-speed grinder; Zhejiang Wenling Linda Machinery Co., Ltd., Wenlin, China) to 0.5 mm particles and stored in desiccators pending further analysis. The content of crude protein (CP) was determined by the Kjeldahl method. The content of neutral detergent fiber (NDF) and acid detergent fiber (ADF) was determined by the Van Soest method [19]. The content of ether extract (EE) was determined by the Soxhlet extraction technique [20]. A pH meter (PHS-3C, China Shanghai Instrument and Electrical Science Instrument Co., Ltd., Shanghai, China) was used to measure the pH. Ammoniacal nitrogen (NH_3_-N) was analyzed using the phenol-hypochlorite program [21]. Organic acids (lactic acid, LA; acetic acid, AA; butyric acid, BA; propionic acid, PA) were quantified using high-performance liquid chromatography [21] (HPLC, 1260 Infinity II, Agilent Technologies, Waldbronn, Germany) equipped with a C18 column (FMF-5559-EONU, 150 × 4.6 mm, 5 μm; FLM Scientific Instrument Co., Ltd., Guangzhou, China). The separation was achieved with a mobile phase consisting of 20 mM potassium dihydrogen phosphate buffer (pH 2.50) and methanol (97:3, *v*/*v*), filtered through a 0.22 μm nylon membrane and degassed by sonication for 15 min. Isocratic elution was performed at 0.6 mL/min with the column oven maintained at 50 °C. Total water-soluble carbohydrates (WSCs) were quantified as glucose equivalents using anthrone-sulfuric acid colorimetry according to AOAC Official Method 977.22 [22]. This measures acid-hydrolyzable saccharides including mono- and disaccharides, with results representing total fermentable carbohydrate potential rather than individual sugar concentrations.

The liquid culture medium and physiological saline were divided into separate conical bottles that were sealed and sterilized under high pressure (121 °C, 15 min). After cooling, the physiological saline solution (90 mL) was mixed with the silage sample (10 g per bag) at a ratio of 9:1, sealed in conical bottles, and incubated in an oscillator at a constant temperature for 30 min. The sample solution was then diluted to a predesigned gradient in a laminar flow cabinet (10 mL culture medium and 1 mL sample solution) and incubated. LAB were cultured in MRS medium at 28 °C for 24 h, while the mold was cultured in high-salt PDA medium at 37 °C for 72 h. The yeast was propagated on malt extract agar medium at 37 °C for 72 h, and the aerobic bacteria was cultured on nutrient agar medium at 28 °C for 24 h. After completing the culture process, the microorganisms were counted [23]. The nutrient composition and microbial counts of the raw silage feedstuffs are listed in Table 1.

### 2.3. Bacterial Community Analysis

An E.Z.N.A.^®^ soil kit was used to extract DNA from feed samples, the quality of DNA was detected using 1% agarose gel electrophoresis, concentration and purity of DNA was determined using a microvolume spectrophotometer, V3–V4 variable region of the 16SrRNA gene was amplified using PCR, and DNA extracted from the feed samples was used as a template. Primers 338F (5′-ACTCCTACGGGAGGCAGCAG-3′) and 806R (5′-GGACTACH VGGGTWTCTAAT-3′) were selected, and the PCR products were subsequently recovered and detected the using 2% agarose gel electrophoresis. A library was built for the purified and detected PCR products, and the Illumina platform was used for sequencing, followed by high-throughput sequencing data analysis [24,25].

### 2.4. Statistical Analysis

Data were collected and analyzed using Microsoft Office Excel 2021. The experiment followed a completely randomized design, and one-way analysis of variance with Duncan’s multiple comparison tests was conducted using IBM SPSS (version 17.0; SPSS Inc., Chicago, IL, USA). Data are presented as the means ± standard error (SE). Where ANOVA indicated significant effects (*p* < 0.05). Microbial count data (LAB, yeast, mold, aerobic bacteria) were log10-transformed to achieve normality and homogeneity of variance. Sequencing data for the bacterial communities were analyzed using the free online Majorbio Cloud Platform (www.Majorbio.com, accessed on 2 Mar 2025).

## 3. Results

### 3.1. Changes in Nutritional Quality of Mulberry Branches and Leaves During Silage Fermentation

The dynamics of the chemical components in the mulberry silage inoculated with *L. plantarum* and cellulase are presented in Table 2 and Table 3. With the extension of silage time, the DM content of each group decreased; however, at the same fermentation time, the DM content of the inoculated groups was significantly higher than that of the Control group (*p* < 0.05). On the 7th d of fermentation, the CP content of the LPC group was lower than that of the LP and Control groups (*p* < 0.05); however, on the 60th d, the CP content of the LPC group was higher than that of the Control and LP groups (*p* < 0.05). The variation in EE content in each group with fermentation time was not obvious; however, the EE content in the inoculation group was higher than that in the Control group. Before fermentation of the silage for 30 d, the NDF content in the raw materials was significantly reduced by inoculation (*p* < 0.05). On the 60th d, the NDF and ADF contents of the Control group decreased, whereas those of the LP and LPC groups increased significantly and were higher than those of the Control group (*p* < 0.05). In addition, the soluble carbohydrate content in each group increased with fermentation time and then decreased.

### 3.2. Changes in Fermentation Quality of Mulberry Branches and Leaves During Silage Fermentation

The dynamics of the fermentation characteristics of the mulberry branch silage are presented in Table 4. Throughout the fermentation process, the pH of the Control and LPC and LP groups showed a downward trend. Especially in the late stage of silage fermentation, the pH of the LPC group was significantly lower than that of the Control and LP groups (*p* < 0.05); however, on the 60th d of fermentation, the pH of the Control increased. In all groups, the LA content increased gradually from the 0th to 30th d, but decreased at the 60th d. On the 0th and 60th d of silage, the LA in the LPC and LP groups was significantly higher than that in the Control group (*p* < 0.05). On the 15th and 30th d of silage, the concentrations of AA and PA in the LPC group were significantly higher than those in the other groups (*p* < 0.05). However, PA was not detected in any of the groups on the 60th d of silage. Moreover, BA was not detected throughout the fermentation period. In addition, the NH_3_-N content of all groups showed an increasing trend throughout the silage process; however, the NH_3_-N content of the inoculation group was significantly higher than that of the Control group (*p* < 0.05), and it decreased in the LPC group on the 60th d.

### 3.3. Change in Microorganism Number During Silage Fermentation of Mulberry Branch and Leaf

The dynamics of the main microorganism counts in mulberry silage are presented in Table 5. The number of LAB subjected to the Control and inoculation treatments initially increased, then decreased throughout the fermentation period, whereas the yeast, mold, and aerobic bacteria counts continued to decrease. Specifically, on the 30th d of silage, the number of LAB in the Control and inoculation groups increased to the highest value, with the number of LAB in the LP group significantly higher than that in the LPC and Control groups (*p* < 0.05). On the 60th d of silage, the LAB count decreased. On the 30th d of silage, the yeast and mold counts in the LP and LPC groups were significantly lower than those in the Control group (*p* < 0.05). In addition, mold growth was significantly inhibited in the LP and LPC groups (*p* < 0.05).

### 3.4. Alpha Diversity Analysis of Mulberry Twig and Leaf Silage and Beta Diversity Analysis

**Figure 1:**
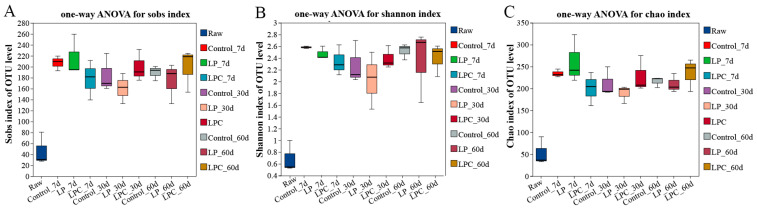
Alpha diversity analysis of mulberry silage. Note: **LP** denotes the addition of *L. plantarum* (5 × 10^6^ CFU·g^−1^ fresh weight)*,* whereas **LPC** represents the addition of *L. plantarum* (5 × 10^6^ CFU·g^−1^ fresh weight) combined with cellulase (0.3% of fresh weight; enzyme activity ≥ 5000 U·g^−1^); Raw, raw material.

Microbial community data were acquired via high-throughput sequencing of mulberry leaf silage samples fermented for 7, 30, and 60 d. The sequencing depth was adequate, with an average effective sequence length of 424 bp, encompassing most species within the microbial community. At a similarity threshold of 97%, all sequenced reads were clustered into 1,190 operational taxonomic units. Analysis of the microbial diversity revealed notable differences between the groups. Figure 1A–C shows the dynamics of the Shannon and Chao indices. Compared with the raw mulberry branches, the microbial diversity and richness of mulberry branches after fermentation were significantly improved.

**Figure 2:**
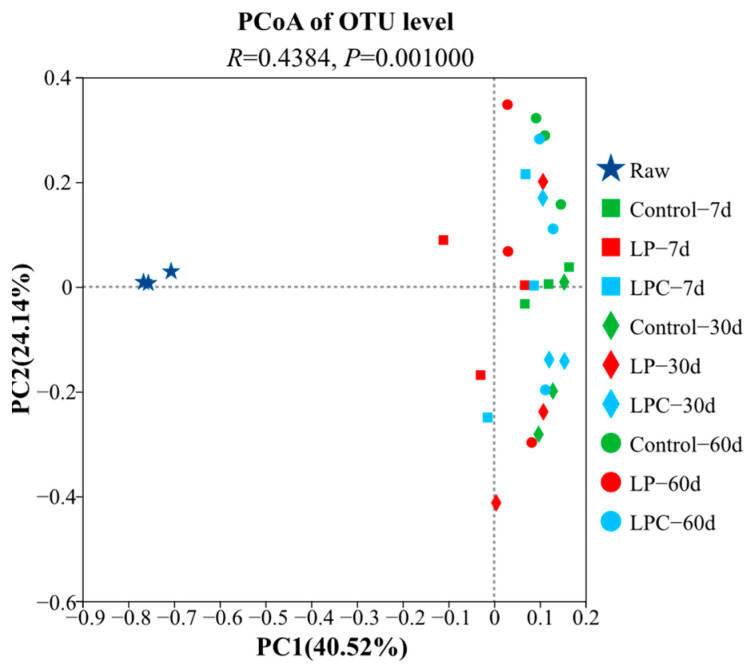
Analysis of the bacterial diversity in mulberry branch silage based on principal coordinate analysis (PCoA). Note: **LP** denotes the addition of *L. plantarum* (5 × 10^6^ CFU·g^−1^ fresh weight)*,* whereas **LPC** represents the addition of *L. plantarum* (5 × 10^6^ CFU·g^−1^ fresh weight) combined with cellulase (0.3% of fresh weight; enzyme activity ≥ 5000 U·g^−1^). Raw, raw material.

Principal coordinate analysis (Figure 2) was used to evaluate the bacterial communities of the mulberry and leaf silage materials in the Control, LP, and LPC groups. The results showed that the distribution of bacterial communities in the LP and LPC groups changed after 60 d of silage (*R* = 0.4384, *p* < 0.01).

### 3.5. Analysis of the Bacterial Community Composition of the Branch and Leaf Silage Microbiome

**Figure 3:**
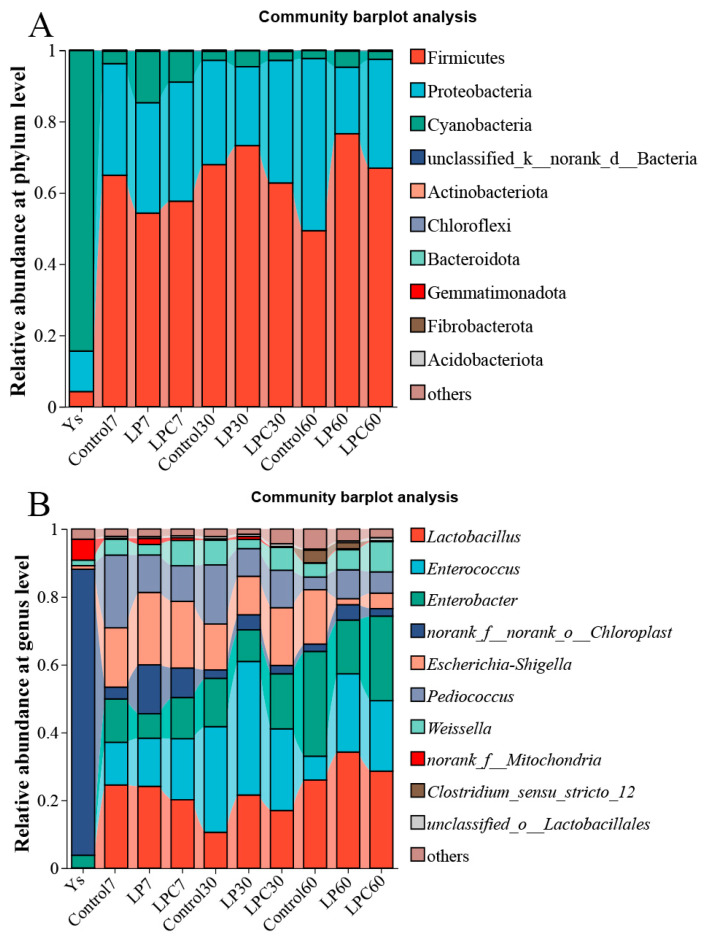
(**A**) Microbial community structure of mulberry leaf silage at the phylum levels. (**B**) Microbial community structure of mulberry leaf silage at the genus levels. Note: **LP** denotes the addition of *L. plantarum* (5 × 10^6^ CFU·g^−1^ fresh weight)*,* whereas **LPC** represents the addition of *L. plantarum* (5 × 10^6^ CFU·g^−1^ fresh weight) combined with cellulase (0.3% of fresh weight; enzyme activity ≥ 5000 U·g^−1^).

Figure 3A illustrates the relative abundance of raw materials and each group after 7, 30, and 60 d of fermentation. The relative abundance of cyanobacteria was the highest in raw silage (84.36%). In all stages of the silage process, Firmicutes was the dominant phylum in all groups (60.74–68.06%), followed by Proteobacteria (23.91–36.30%). At the genus level (Figure 3B), the relative abundance of *Norank_f_norank_o_Chloroplast* was the highest (84.36%), followed by *Norank_f_mitochondria* (6.15%) and *Enterobacter* (3.74%). On the 7th d, the dominant bacteria in each group were *Lactiplantibacillus* (20.17–24.55%), followed by *Escherichia-Shigella* (17.49–21.34%). On the 30th d, the dominant bacteria were *Enterococcus* (24.08–39.37%), and on the 60th d, the dominant bacteria were *Lactiplantibacillus* (25.98–34.28%), *Enterobacter* (15.86–30.90%), and *Enterococcus* (7.03–23.07%).

**Figure 4:**
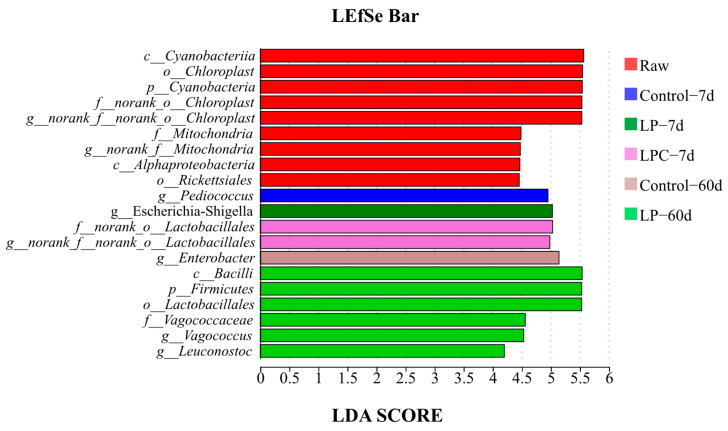
LEfSe analysis of mulberry foliage silage. Note: **LP** denotes the addition of *L. plantarum* (5 × 10^6^ CFU·g^−1^ fresh weight)*,* whereas **LPC** represents the addition of *L. plantarum* (5 × 10^6^ CFU·g^−1^ fresh weight) combined with cellulase (0.3% of fresh weight; enzyme activity ≥ 5000 U·g^−1^); Raw, raw material.

LEfSe was performed to further explore variations in the bacterial communities among the groups (Figure 4). On the 7th d of ensiling, the relative abundance of *Pediococcus* in the Control group, *Escherichia-Shigella* in the LP group, and two norank strains of *Lactiplantibacillus* (*norank_o__Lactiplantibacillus* and *norank_f__norank_o__Lactiplantibacillus*) in the LPC group were higher (*p* < 0.05). On the 60th d of ensiling, the relative abundances of *Enterobacter* in the Control group and *Vagocococcus* and *leuconostoc* in the LP group were significantly higher (*p* < 0.05).

**Figure 5:**
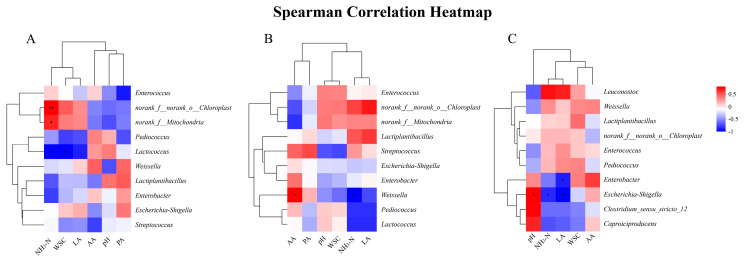
(**A**) Correlation analysis between the microbial community (genus level) and fermentation characteristics of mulberry branch silage on day 7. (**B**) Correlation analysis between the microbial community (genus level) and fermentation characteristics of mulberry branch silage on day 30. (**C**) Correlation analysis between the microbial community (genus level) and fermentation characteristics of mulberry branch silage on day 60. Note: NH_3_-N, ammoniacal nitrogen; WSC, water-soluble carbohydrate; LA, lactic acid; AA, acetic Acid; PA, propanoic acid. “*” indicates significant correlation (*p* < 0.05); “**” indicates highly significant correlation (*p* < 0.01).

Figure 5 presents Spearman correlation heatmaps between the VIF-screened fermentation parameters (LA, AA, PA, CP, and NH_3_-N) and top 10 microbial genera during silage fermentation on days 7 (5A), 30 (5B), and 60 (5C). Correlation analysis between the top 10 bacteria with the highest abundance and fermentation index of mulberry branches was carried out according to silage time. On the 7th d of silage, the NH_3_-N content was correlated with *norank_f__norank_o__Chloroplast* (*R* = 0.82, *p* < 0.01) and *norank_f__Mitochondria* (*R* = 0.7, *p* < 0.01). A significant correlation was noted between the WSC content and *Lactococcus* (*R* = −0.7, *p* < 0.05). On the 30th d of silage, *Weissella* was significantly correlated with the AA content *(R* = 0.72, *p* < 0.05) and NH_3_-N content (*R* = −0.78, *p* < 0.05). On the 60th d of silage, a significant correlation was determined between *Enterobacter* and LA content (*R* = −0.72, *p* < 0.05), between *Escherichia-Shigella* and the pH value (*R* = 0.68, *p* < 0.05), and NH_3_-N content (*R* = −0.68, *p* < 0.05) and LA content.

## 4. Discussion

### 4.1. Effect of L. plantarum and Cellulase on the Nutritional Quality, Fermentation Characteristics, and Microorganism Count of Mulberry Silage

The observed reduction in DM loss during the initial phase (before day 30) in *L. plantarum* inoculated groups (LP and LPC) compared to the Control (Table 2) can be primarily attributed to the rapid establishment of an anaerobic, acidic environment facilitated by the exogenous LAB [26]. Similar findings by Li et al. [14] underscore the role of LAB inoculants in suppressing aerobic respiration and undesirable microbial activity that drive DM loss. Microorganisms attached to silage raw materials need to use carbon sources for their growth activities, leading to a decrease in NDF and ADF in the early stages of silage [15], consistent with the conclusion of this study. The NDF and ADF contents initially decreased, then increased because the LP group effectively alleviated fiber degradation in the later stages of silage fermentation [19], thus maintaining the fiber content. The significant increase in WSC content during the early fermentation phase was primarily driven by the rapid hydrolysis of structural carbohydrates (e.g., cellulose and hemicellulose) by plant-derived enzymes [27]. The comparatively slower rate of WSC decline in the LPC group towards the end of ensiling might indicate either a sustained, albeit slower, release of sugars from ongoing cellulase activity or a more efficient microbial community structure better adapted to utilizing complex substrates, contributing to prolonged fermentation stability [27,28].

The rapid and sustained production of LA and AA, particularly in the inoculated groups (Table 4), is the core driver of pH reduction and the key mechanism for inhibiting some of spoilage microorganisms (yeast, mold, Table 5) and preserving silage quality [29,30]. Moreover, the pH of the LPC group decreased more during the fermentation process, indicating that the combined addition of *L. plantarum* and cellulase was helpful for maintaining fibrous substances in mulberry leaves, inhibiting miscellaneous bacteria, and reducing CP loss and NH_3_-N production in silage [5]. On the 7th d of silage fermentation, the LA content rapidly increased, attributed to the increase in LA content during the initial stage caused by inoculation with LAB [31,32]. The sustained dominance of acetic acid production in the LPC group, particularly evident after 15th d, suggests that the combined inoculant promoted a shift towards heterofermentative metabolism within the mulberry silage system [33]. As is well known, the main product of homogeneous fermentation is LA, whereas that of heterogeneous fermentation is AA and LA. This is consistent with the increasing trend in the relative abundance of *Enterobacter* during the later stages of fermentation in this study. *Enterobacter* is generally considered to be more inclined towards heterogeneous fermentation [34]. PA also showed a similar trend; however, the undetectable PA at 60 d likely resulted from biological consumption by dominant *Lactiplantibacillus* utilizing PA as an auxiliary energy source amid declining WSC (WSC% DM: decreased from 6.15 to 3.94 in Control; Table 2) [35]. In addition, the disappearance of PA may involve active reutilization by LAB as an alternative carbon source, consistent with recent studies demonstrating that *Lactiplantibacillus* spp. can metabolize short-chain fatty acids (including PA) under energy-limited conditions [35]. The LP and LPC groups effectively maintained an acidic anaerobic fermentation environment and inhibited yeast and mold growth. In summary, the inoculation of *L. plantarum* alone and in combination with cellulase played a key role in supporting the reduction in DM loss, maintenance of fiber content, and improvement of overall feed quality of mulberry branches.

### 4.2. Effect of L. plantarum and Cellulase on the Bacterial Community Composition of Mulberry Branch Leaf Silage Fermentation

The minimal divergence in bacterial community composition between the Control and inoculated groups throughout fermentation (7–60 d) suggests that the native microbiota of mulberry branches, when subjected to anaerobic conditions, inherently possesses sufficient functional capacity to initiate and sustain ensiling without exogenous augmentation [36]. The predominance of *Lactiplantibacillus* in LP and LPC treatments during early fermentation drove rapid acidification through efficient lactic acid production, creating a low-pH environment that suppressed spoilage microorganisms [37]. In the Control group, early dominance of facultative anaerobes like *Pediococcus* likely facilitated initial oxygen scavenging and acid production prior to strict anaerobe establishment [38]. Inoculation with *L. plantarum* in LP/LPC groups preemptively occupied this niche, suppressing *Pediococcus* through competitive exclusion. The transient dominance of *Enterococcus* at mid-ensiling (30th d) across all groups may reflect its tolerance to moderately acidic conditions. The pH of the silage system decreased slowly at this stage, and the slightly acidic environment did not inhibit the growth of *Enterococcus*, which promoted the dominance of *Enterococcus* [39]. By day 60, community succession favored acid-tolerant *Lactiplantibacillus* and versatile *Enterobacter*, indicating adaptation to stable acidic and nutrient-limited conditions [40]. Certain *Enterobacter* species are facultative anaerobes capable of utilizing diverse carbon sources, potentially contributing to AA production (Table 4) [41]. Although *Enterobacter* was highly abundant in the late stage of silage (e.g., 30.90% in Control at day 60). However, their proliferation may also indicate suboptimal fermentation conditions, as *Enterobacter* can compete with LAB for substrates and produce NH_3_-N through proteolysis, leading to elevated pH and nutrient loss [42]. The butyric acid (BA) was detected in all groups and the significantly lower NH_3_-N in LPC (0.82% vs. 0.88% in LP and 0.55% in Control) suggest that spoilage was effectively suppressed. The combined inoculation (LPC) reduced *Enterobacter* abundance by 49% compared to Control (15.86% vs. 30.90%), likely due to competitive exclusion by dominant *Lactiplantibacillus* and accelerated acidification (Figure 3B, Table 4). Nevertheless, future studies should monitor *Enterobacter* dynamics during aerobic exposure to assess its implications for silage stability post-opening.

In addition, with increasing fermentation time, the acidic environment of the silage system tended to be stable, and *Lactiplantibacillus* was still the dominant flora in the silage fermentation systems. Thus, it played an important role and continued to produce lactic acid to maintain the acidic environment, thus ensuring fermentation quality [39]. The metabolism of the bacterial community greatly influenced the fermentation index during silage fermentation. Despite low abundance, *Lactococcus* showed significant negative correlation with NH_3_-N accumulation (Figure 5), suggesting synergistic interactions with dominant *Lactiplantibacillus* in suppressing proteolysis, possibly through competitive exclusion of ammonifying bacteria [43]. In this study, after 60 d of silage, the pH and NH_3_-N content were negatively correlated with *Enterobacter*. In addition, the LA content was significantly negatively correlated with *Escherichia-Shigella*, especially in the late stage of fermentation, and the inoculation of *L. plantarum* significantly inhibited the activities of harmful microorganisms such as *Escherichia-Shigella* in the mulberry silage system.

## 5. Conclusions

This study investigated the synergistic effects of *L. plantarum* (LP) and cellulase on mulberry silage quality and microbial dynamics over 60-day ensiling. Key findings demonstrate that combined inoculation (LPC group) significantly enhanced nutritional preservation (reduced DM loss by 6.3% vs. Control; increased CP by 7.1% at day 60), accelerated acidification (pH 4.64 vs. 5.44 in Control; elevated LA by 29.4%), and stabilized microbial ecology (the relative abundance of *Lactiplantibacillus* (71.63–83.28%) remained at a high level, effectively inhibiting the growth of mold, aerobic bacteria, and *Enterobacteria* (0.08–1.14%)). Methodological limitations include the absence of a cellulase-only group, which precludes precise quantification of its individual contribution to fiber degradation. Nevertheless, our results robustly demonstrate that combining *L. plantarum* with cellulase (LPC) maximizes fermentation efficiency, nutritional preservation, and microbial stability in mulberry silage.

## Figures and Tables

**Table 1:** Characteristics of the mulberry branches and leaves.

Index	Content
DM/%	50.35 ± 0.82
CP/% DM	14.66 ± 0.28
NDF/% DM	54.30 ± 1.04
ADF/% DM	27.06 ± 0.47
EE/% DM	5.92 ± 0.05
WSC/% DM	8.38 ± 0.19
LAB/logCFU·g^−1^ FM	5.24 ± 0.06
Yeast/log CFU·g^−1^ FM	4.74 ± 0.04
Mold/logCFU·g^−1^ FM	2.83 ± 0.02
AB/logCFU·g^−1^ FM	8.63 ± 0.04

DM, dry matter; CP, crude protein; NDF, neutral detergent fiber; ADF, acid detergent fiber; EE, ether extract; WSC, water-soluble carbohydrates; LAB, lactic acid bacteria; AB, aerobic bacteria; FM, fresh weight. Data are presented as the means ± standard error.

**Table 2:** Effect of *L. plantarum* and cellulase inoculation on the Chemical Composition of mulberry branch and leaf silage.

Index	Time	Groups			*p*-Value		
		Control	LP	LPC	Groups	Time	G × T
DM%	7 d	45.88 ± 0.25 ^Ba^	47.35 ± 0.27 ^Aa^	48.26 ± 1.05 ^Aa^	0.0001	0.0001	0.2183
	15 d	44.02 ± 0.38 ^Bb^	44.97 ± 0.20 ^Bb^	46.44 ± 1.10 ^Ab^			
	30 d	41.49 ± 0.61 ^Bc^	42.37 ± 0.67 ^Bc^	45.43 ± 0.76 ^Ab^			
	60 d	40.58 ± 0.69 ^Bc^	41.62 ± 0.85 ^Bc^	43.15 ± 0.37 ^Ac^			
CP% DM	7 d	14.55 ± 0.11 ^Aa^	14.31 ± 0.04 ^Aa^	13.29 ± 0.06 ^Bb^	0.0264	0.2682	0.0001
	15 d	13.64 ± 0.02 ^Ab^	12.71 ± 0.06 ^Bb^	12.38 ± 0.08 ^Cc^			
	30 d	13.77 ± 0.04 ^Ab^	12.15 ± 0.03 ^Bc^	12.22 ± 0.20 ^Bc^			
	60 d	12.89 ± 0.18 ^Bc^	12.92 ± 0.24 ^Bb^	13.8 ± 0.12 ^Aa^			
EE% DM	7 d	4.02 ± 0.22 ^Ac^	4.33 ± 0.33 ^Ac^	4.05 ± 0.09 ^Ac^	0.0354	0.4521	0.6452
	15 d	5.91 ± 0.25 ^Aa^	6.45 ± 0.39 ^Aa^	6.54 ± 0.21 ^Aa^			
	30 d	5.49 ± 0.59 ^Aab^	5.68 ± 0.53 ^Ab^	5.94 ± 0.26 ^Aa^			
	60 d	4.88 ± 0.08 ^Ab^	5.17 ± 0.10 ^Ab^	5.11 ± 0.16 ^Ab^			
WSC% DM	7 d	3.23 ± 0.16 ^Bb^	3.95 ± 0.17 ^Ab^	4.52 ± 1.02 ^Ab^	0.2958	0.0003	0.0253
	15 d	5.9 ± 0.13 ^Aa^	6.27 ± 0.15 ^Aa^	6.36 ± 0.28 ^Aa^			
	30 d	6.15 ± 0.28 ^Aa^	6.15 ± 0.07 ^Aa^	5.8 ± 0.17 ^Aa^			
	60 d	3.94 ± 0.17 ^Ab^	3.97 ± 0.24 ^Ab^	4.2 ± 0.16 ^Ab^			

Note: **LP** denotes the addition of *L. plantarum* (5 × 10^6^ CFU·g^−1^ fresh weight)*,* whereas **LPC** represents the addition of *L. plantarum* (5 × 10^6^ CFU·g^−1^ fresh weight) combined with cellulase (0.3% of fresh weight; enzyme activity ≥ 5000 U·g^−1^). The same index within the same column, marked with different lowercase letters (e.g., a, b, c, d), denotes statistically significant differences between fermentation days under the same treatment; capital letters (e.g., A, B, C) within the same row denote significant differences between groups on the same fermentation day(s). DM, dry matter; CP, crude protein; EE, ether extract; WSC, water-soluble carbohydrate. Data are presented as the means ± standard error.

**Table 3:** Effect of *L. plantarum* and cellulase inoculation on the fiber degradation of mulberry branch and leaf silage.

Index	Time	Groups			*p*-Value		
		Control	LP	LPC	Groups	Time	G × T
NDF% DM	7 d	46.77 ± 1.27 ^Aa^	45.75 ± 0.95 ^Aa^	42.01 ± 1.63 ^Bab^	0.0426	0.5734	0.0001
	15 d	46.96 ± 0.79 ^Aa^	41.31 ± 2.61 ^Bb^	35.05 ± 1.76 ^Cc^			
	30 d	46.48 ± 1.00 ^Aa^	41.19 ± 1.06 ^Bb^	40.06 ± 1.64 ^Bb^			
	60 d	41.45 ± 0.73 ^Bb^	43.74 ± 0.96 ^Ab^	44.97 ± 0.72 ^Aa^			
ADF% DM	7 d	25.93 ± 1.18 ^Aa^	23.34 ± 1.18 ^Ba^	20.32 ± 0.84 ^Cb^	0.0105	0.5921	0.0103
	15 d	25.74 ± 1.41 ^Aa^	23.42 ± 0.89 ^Ba^	20.69 ± 0.71 ^Cb^			
	30 d	26.83 ± 0.51 ^Aa^	21.27 ± 0.69 ^Ba^	21.08 ± 1.17 ^Bb^			
	60 d	25.45 ± 0.47 ^Aa^	23.61 ± 1.02 ^Aa^	24.39 ± 0.83 ^Aa^			

Note: **LP** denotes the addition of *L*. (5 × 10^6^ CFU·g^−1^ fresh weight)*,* whereas **LPC** represents the addition of *L. plantarum* (5 × 10^6^ CFU·g^−1^ fresh weight) combined with cellulase (0.3% of fresh weight; enzyme activity ≥ 5000 U·g^−1^). The same index within the same column, marked with different lowercase letters (e.g., a, b, c), denotes statistically significant differences between fermentation days under the same treatment; capital letters (e.g., A, B, C) within the same row denote significant differences between groups on the same fermentation day(s). NDF, neutral detergent fiber; ADF, acid detergent fiber. Data are presented as the means ± standard error.

**Table 4:** Changes in fermentation quality during mulberry branch and leaf silage.

Index	Time	Groups			*p*-Value		
		Control	LP	LPC	Groups	Time	G × T
pH	7 d	6.21 ± 0.18 ^Aa^	5.38 ± 0.03 ^Ba^	5.02 ± 0.02 ^Ca^	0.0149	0.0798	0.0001
	15 d	5.41 ± 0.18 ^Ab^	5.27 ± 0.02 ^Aab^	4.9 ± 0.02 ^Bab^			
	30 d	5.04 ± 0.04 ^Ac^	5.04 ± 0.04 ^Ac^	4.8 ± 0.06 ^Bbc^			
	60 d	5.44 ± 0.12 ^Ab^	5.09 ± 0.02 ^Bbc^	4.64 ± 0.13 ^Cc^			
LA/(g·kg^−1^ DM)	7 d	1.64 ± 0.04 ^Bc^	2.17 ± 0.03 ^Ac^	2.24 ± 0.04 ^Ac^	0.0161	0.0006	0.0001
	15 d	2.64 ± 0.18 ^Bb^	3.81 ± 0.07 ^Aa^	3.75 ± 0.23 ^Aa^			
	30 d	3.5 ± 0.04 ^Ba^	3.82 ± 0.13 ^Aa^	3.79 ± 0.10 ^Aa^			
	60 d	2.69 ± 0.18 ^Bb^	3.34 ± 0.28 ^Ab^	3.48 ± 0.15 ^Ab^			
AA/(g·kg^−1^ DM)	7 d	1.29 ± 0.08 ^ABa^	1.07 ± 0.11 ^Ba^	1.42 ± 0.06 ^Aab^	0.0398	0.1084	0.0001
	15 d	1.04 ± 0.14 ^Bab^	1.11 ± 0.11 ^Ba^	1.45 ± 0.14 ^Aab^			
	30 d	0.93 ± 0.11 ^Bbc^	0.63 ± 0.03 ^Bb^	1.63 ± 0.37 ^Aa^			
	60 d	0.64 ± 0.04 ^Bc^	0.37 ± 0.04 ^Bb^	1.22 ± 0.26 ^Ab^			
PA/(g·kg^−1^ DM)	7 d	0.12 ± 0.01 ^Ab^	0.12 ± 0.03 ^Ab^	0.14 ± 0.04 ^Ab^	0.0552	0.0127	0.0051
	15 d	0.22 ± 0.02 ^Ba^	0.24 ± 0.01 ^Ba^	0.35 ± 0.02 ^Aa^			
	30 d	0.23 ± 0.05 ^Ba^	0.24 ± 0.02 ^Ba^	0.37 ± 0.03 ^Aa^			
	60 d	ND	ND	ND			
NH_3_-N/TN%DM	7 d	0.36 ± 0.03 ^Bb^	0.55 ± 0.02 ^Ac^	0.54 ± 0.04 ^Ac^	0.0009	0.0049	0.007
	15 d	0.38 ± 0.01 ^Cb^	0.55 ± 0.12 ^Bc^	0.7 ± 0.01 ^Ab^			
	30 d	0.39 ± 0.02 ^Cb^	0.68 ± 0.01 ^Bb^	0.84 ± 0.02 ^Abc^			
	60 d	0.55 ± 0.05 ^Ba^	0.88 ± 0.07 ^Aa^	0.82 ± 0.02 ^Aa^			

Note: **LP** denotes the addition of *L. plantarum* (5 × 10^6^ CFU·g^−1^ fresh weight)*,* whereas **LPC** represents the addition of *L. plantarum* (5 × 10^6^ CFU·g^−1^ fresh weight) combined with cellulase (0.3% of fresh weight; enzyme activity ≥ 5000 U·g^−1^). The same index within the same column marked with different lowercase letters (e.g., a, b, c), denotes statistically significant differences between fermentation days under the same treatment; capital letters (e.g., A, B, C) within the same row denote significant differences between groups on the same fermentation day(s). LA, lactic acid; AA, acetic Acid; PA, propanoic acid; NH_3_-N, ammoniacal nitrogen; DM, dry matter. Data are presented as the means ± standard error.

**Table 5:** Changes in microbial quantity during fermentation of mulberry branch and leaf silage.

Index	Time	Groups			*p*-Value		
		Control	LP	LPC	Groups	Time	G × T
LAB (logCFU·g^−1^FM)	7 d	6.93 ± 0.04 ^Bb^	7.34 ± 0.07 ^Ab^	7.04 ± 0.02 ^Bb^	0.0026	0.0004	0.002
	15 d	6.85 ± 0.08 ^Ab^	6.9 ± 0.18 ^Ac^	6.65 ± 0.12 ^Ac^			
	30 d	7.5 ± 0.02 ^Ba^	7.81 ± 0.21 ^Aa^	7.75 ± 0.17 ^ABa^			
	60 d	6.45 ± 0.25 ^Ac^	6.44 ± 0.05 ^Ad^	5.92 ± 0.20 ^Bd^			
Yeast (logCFU/gFM)	7 d	4.68 ± 0.12 ^Aa^	4.59 ± 0.09 ^Aa^	4.34 ± 0.05 ^Ba^	0.0001	0.0001	0.9509
	15 d	4.44 ± 0.06 ^Aab^	4.37 ± 0.1 ^Aa^	4.21 ± 0.06 ^Aa^			
	30 d	4.35 ± 0.12 ^Aab^	3.98 ± 0.09 ^Bb^	3.8 ± 0.12 ^Bb^			
	60 d	3.74 ± 0.08 ^Ac^	3.63 ± 0.22 ^ABc^	3.4 ± 0.16 ^Bc^			
Mold (logCFU/gFM)	7 d	2.35 ± 0.05 ^Aa^	2.12 ± 0.06 ^Ba^	1.8 ± 0.01 ^Ca^	0.0104	0.0001	0.0008
	15 d	1.69 ± 0.20 ^Ab^	1.37 ± 0.19 ^Bb^	1.21 ± 0.02 ^Bb^			
	30 d	1.00 ± 0.11 ^Ac^	0.41 ± 0.14 ^Bc^	0.33 ± 0.09 ^Bc^			
	60 d	0.23 ± 0.04 ^Ad^	0.22 ± 0.04 ^Ac^	0.10 ± 0.03 ^Ad^			
Aerobic bacteria(logCFU/gFM)	7 d	8.22 ± 0.06 ^Aa^	8.13 ± 0.05 ^Aa^	7.97 ± 0.10 ^Aa^	0.0649	0.0001	0.4839
	15 d	7.71 ± 0.14 ^Ab^	7.43 ± 0.1 ^Ab^	7.51 ± 0.34 ^Ab^			
	30 d	7.38 ± 0.09 ^Ab^	7.12 ± 0.06 ^Ab^	7.09 ± 0.10 ^Ac^			
	60 d	6.62 ± 0.40 ^Ac^	6.72 ± 0.15 ^Ac^	6.55 ± 0.06 ^Ad^			

Note: **LP** denotes the addition of *L. plantarum* (5 × 10^6^ CFU·g^−1^ fresh weight)*,* whereas **LPC** represents the addition of *L. plantarum* (5 × 10^6^ CFU·g^−1^ fresh weight) combined with cellulase (0.3% of fresh weight; enzyme activity ≥ 5000 U·g^−1^). The same index within the same column marked with different lowercase letters (e.g., a, b, c) denotes significant differences between fermentation days under the same treatment; capital letters (e.g., A, B, C) within the same row denote statistically significant differences between groups on the same fermentation day(s). LAB, lactic acid bacteria; FM, fresh weight. Data are presented as the means ± standard error.

## Data Availability

The data presented in this study are available upon request from the first author. Data supporting the bacterial community in the Sequence Read Archive (SRA) can be found at the National Center for Biotechnology Information (NCBI). SRA number: PRJNA1266670.

## Abbreviations

The following abbreviations are used in this manuscript:

ADF	Acid detergent fiber
CP	Crude protein
EE	Ether extract
DM	Dry matter
LAB	Lactic acid bacteria
LP	*Lactiplantibacillus plantarum*
LPC	*Lactiplantibacillus plantarum* and cellulase
NDF	Neutral detergent fiber
NH_3_-N	Ammoniacal nitrogen
WSC	Water-soluble carbohydrate
